# Recurrent dysplasia epiphysealis hemimelica of the ankle in a child requiring three surgical excisions over five years

**DOI:** 10.1093/jscr/rjag430

**Published:** 2026-06-03

**Authors:** Andrew Kerwin, Danil Chernov, Nicholas Frappa, Morgan Dillon, Samuel Fuller, Matthew G Alben, Jeremy P Doak

**Affiliations:** Jacobs School of Medicine and Biomedical Sciences, Department of Orthopaedics and Sports Medicine, 955 Main Street, Buffalo, NY 14203, United States; Jacobs School of Medicine and Biomedical Sciences, Department of Orthopaedics and Sports Medicine, 955 Main Street, Buffalo, NY 14203, United States; Jacobs School of Medicine and Biomedical Sciences, Department of Orthopaedics and Sports Medicine, 955 Main Street, Buffalo, NY 14203, United States; Jacobs School of Medicine and Biomedical Sciences, Department of Orthopaedics and Sports Medicine, 955 Main Street, Buffalo, NY 14203, United States; Department of Orthopaedics and Sports Medicine, University at Buffalo, 100 High Street, Buffalo, NY 14203, United States; Department of Orthopaedics and Sports Medicine, University at Buffalo, 100 High Street, Buffalo, NY 14203, United States; Department of Orthopaedics and Sports Medicine, University at Buffalo, 100 High Street, Buffalo, NY 14203, United States

**Keywords:** dysplasia epiphysealis hemimelica, Trevor’s disease, childhood neoplasia, orthopaedic oncology, childhood tumor

## Abstract

Dysplasia epiphysealis hemimelica, also known as Trevor’s disease, is a rare developmental disorder characterized by asymmetric epiphyseal osteochondral overgrowth, most commonly affecting the ankle or knee in pediatric patients. Surgical excision is typically indicated when lesions cause deformity, mechanical symptoms, or functional limitation, although recurrence has been reported. We present the case of a three-year-old male with dysplasia epiphysealis hemimelica of the ankle who experienced two recurrences over a five-year period despite apparently complete surgical excision confirmed intraoperatively. The patient ultimately required three operative procedures due to progressive gait disturbance, stiffness, and pain with ambulation. This case demonstrates the potential for repeated recurrence in skeletally immature patients and highlights the importance of symptom-guided management and long-term surveillance in pediatric patients with dysplasia epiphysealis hemimelica.

## Introduction

Dysplasia epiphysealis hemimelica (DEH) is a non-hereditary developmental disorder characterized by unilateral epiphyseal osteochondral overgrowth, most commonly affecting the lower extremity [1–3] , although involvement of the upper extremity has also been reported [4, 5]. DEH has an estimated incidence of ~1 per 1 000 000, with typical onset between two and eight years of age and a reported male predominance [6]. Unlike conventional osteochondromas, which arise from the metaphysis, DEH lesions originate from the epiphysis [7]. The pathogenesis is not fully understood but is thought to involve abnormal cartilage proliferation during early skeletal development, possibly related to disruption of the apical ectodermal cap [8]. Azouz *et al*. classified DEH into three forms: localized (involving a single epiphysis), classic (involving multiple epiphyses within the same limb), and generalized (involving the entire lower extremity) [9].

The clinical presentation of DEH is often non-specific and may include pain with activity, impaired gait mechanics, limited joint motion, and visible deformity from mass effect [6]. Initial diagnosis is typically made with radiographs demonstrating an epiphyseal osteocartilaginous mass, while computed tomography and magnetic resonance imaging (MRI) are useful for surgical planning and evaluation of surrounding soft tissue and osseous structures [10]. Histologic findings are not specific and may resemble isolated osteochondroma, while genetic testing usually demonstrates normal expression of EXT1 and EXT2 tumor suppressor genes, helping differentiate DEH from hereditary multiple exostoses [11]. There is no effective medical therapy, and surgical excision of symptomatic lesions and loose bodies remains the mainstay of treatment to prevent progressive deformity and degenerative joint changes [2, 12]. A recent review of 70 patients by Artioli *et al*. reported recurrence in ~9% of cases following surgical excision [2].

Despite growing awareness of DEH, there remains no clear consensus regarding long-term management or the duration of follow-up, particularly in skeletally immature patients. While recurrence following excision has been reported, guidance on surveillance until skeletal maturity remains limited. We present a pediatric case of classic ankle DEH with two recurrences over five years requiring three surgical excisions, highlighting the potential for repeated osteochondral proliferation prior to skeletal maturity and the need for careful longitudinal follow-up and patient counseling.

## Case report

### Initial presentation

**Figure 1:**
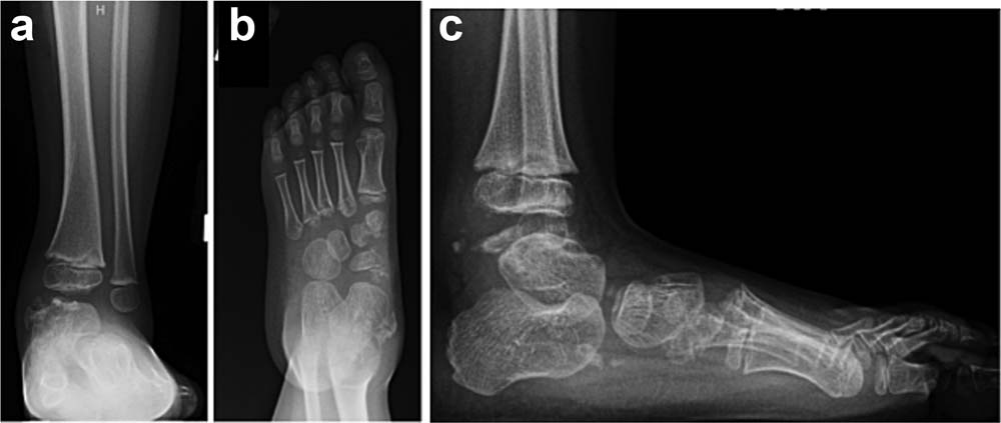
AP ankle (a), AP foot (b), and lateral ankle (c) radiographs demonstrating osteochondral irregularity of the talar dome with disruption of the ankle mortise and flattening of the medial longitudinal arch.

A three-year-old male presented to clinic for evaluation of abnormal appearance of the left foot. Examination revealed in toeing, tibial torsion, hindfoot valgus, pes planus, and a palpable bony prominence along the medial malleolus. He had no significant past medical history. Radiographs of the left ankle demonstrated osteochondral irregularities involving the medial talar dome and medial cuneiform (Fig. 1). MRI further identified five osteochondromas: three arising from the medial talus and two from the medial navicular, confirming the diagnosis of DEH.

### Initial surgical treatment

Surgical excision was performed through a curvilinear anteromedial ankle incision. Osteochondromas from the talus and navicular were removed, and fluoroscopy confirmed complete resection. An additional distal tibial epiphyseal osteochondroma not seen on preoperative imaging was identified and excised intraoperatively. The postoperative course was uncomplicated aside from mild wound dehiscence.

### First recurrence

**Figure 2:**
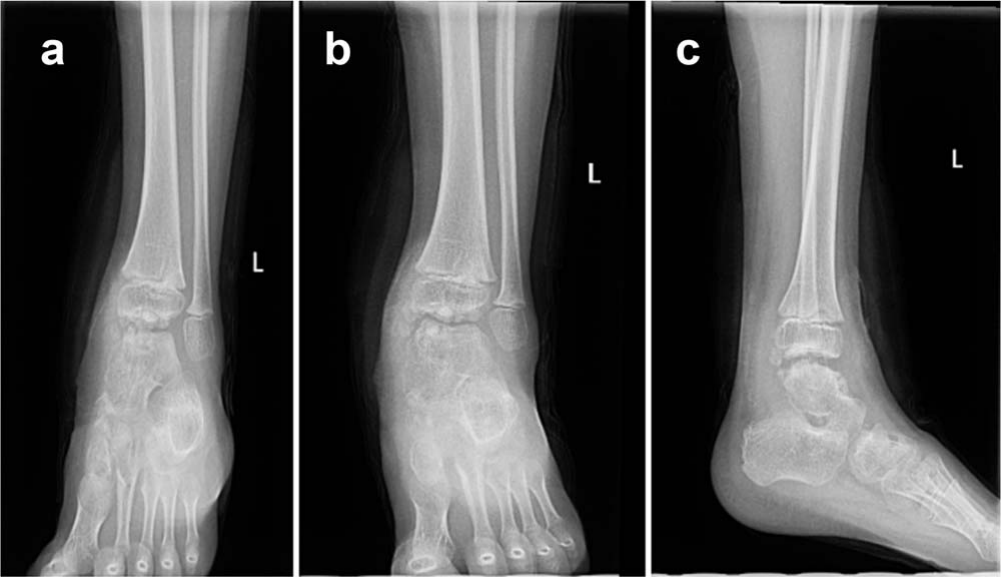
Preoperative AP (a), mortise (b), and lateral (c) ankle radiographs obtained prior to the second surgery demonstrating osteochondromas and irregularity involving the talar dome and distal tibia.

At 9-month follow-up the patient remained asymptomatic, although radiographs demonstrated recurrent ossification along the talus. Pes planus and hindfoot valgus persisted with weightbearing, and management consisted of observation. Fifteen months after the index procedure, the patient returned with pain during ambulation and a limping gait. Imaging demonstrated progression of osteochondral growth involving the talus and distal tibia with intra-articular loose bodies in the tibiotalar joint (Fig. 2).

### Second surgical treatment

A second excision was performed 18 months after the index procedure. An anterior approach was used to remove talar osteochondromas and a loose osseous body, while a posterior curvilinear incision allowed removal of additional lesions from the posterior talus. Fluoroscopy confirmed complete resection. Distal tibial osteochondromas identified on imaging were not excised because they were not producing mechanical symptoms and resection near the distal tibial physis carries risk of growth disturbance in skeletally immature patients. The patient recovered without complications.

### Second recurrence

**Figure 3:**
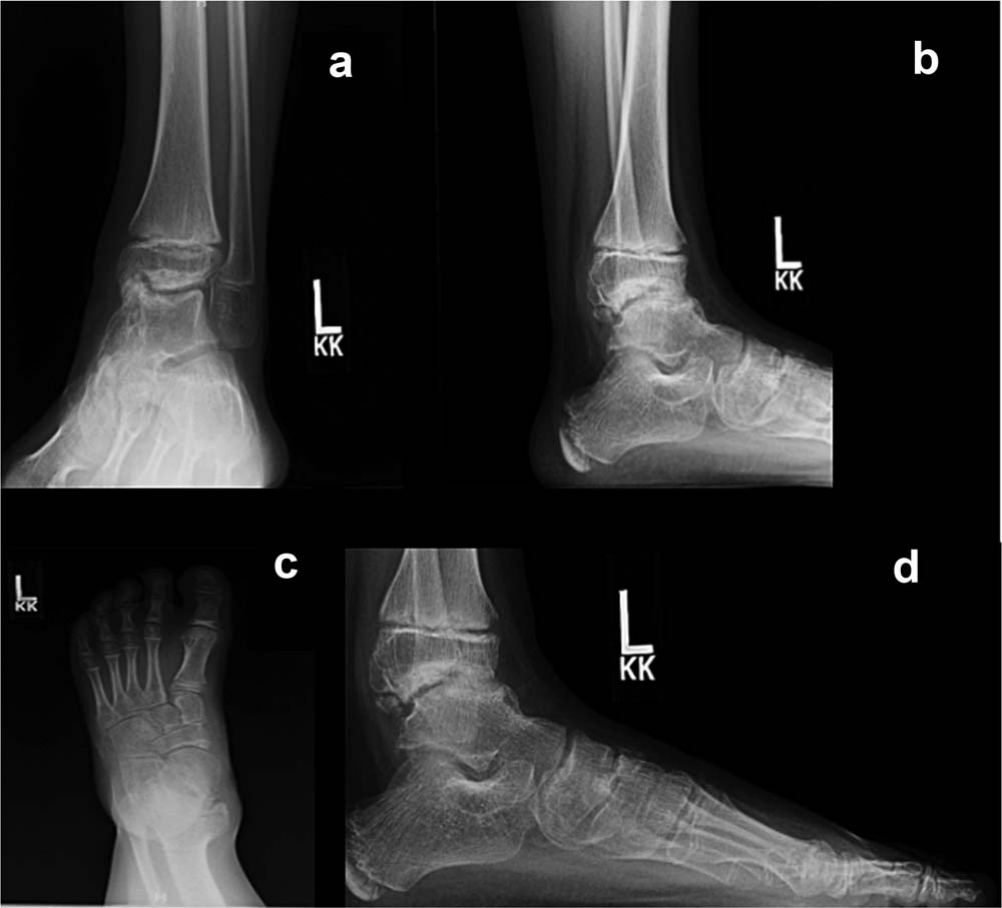
Preoperative AP and lateral ankle (a and b) and foot (c and d) radiographs demonstrating progression of DEH with talar osteochondromas and irregularity of the navicular–medial cuneiform articulation.

Approximately three years after the index procedure, the patient demonstrated gait abnormalities including decreased subtalar motion and persistent pes planovalgus deformity but remained active without functional limitation. Serial radiographs demonstrated progressive osteochondromas involving the talus and talonavicular joint with ossification of the medial cuneiform and medial malleolus (Fig. 3). Given minimal symptoms, management remained observational. Approximately five years after the initial surgery, the patient developed worsening ankle stiffness and dorsal ankle pain with functional limitation, prompting consideration of repeat surgery.

### Third surgical treatment and outcome

**Figure 4:**
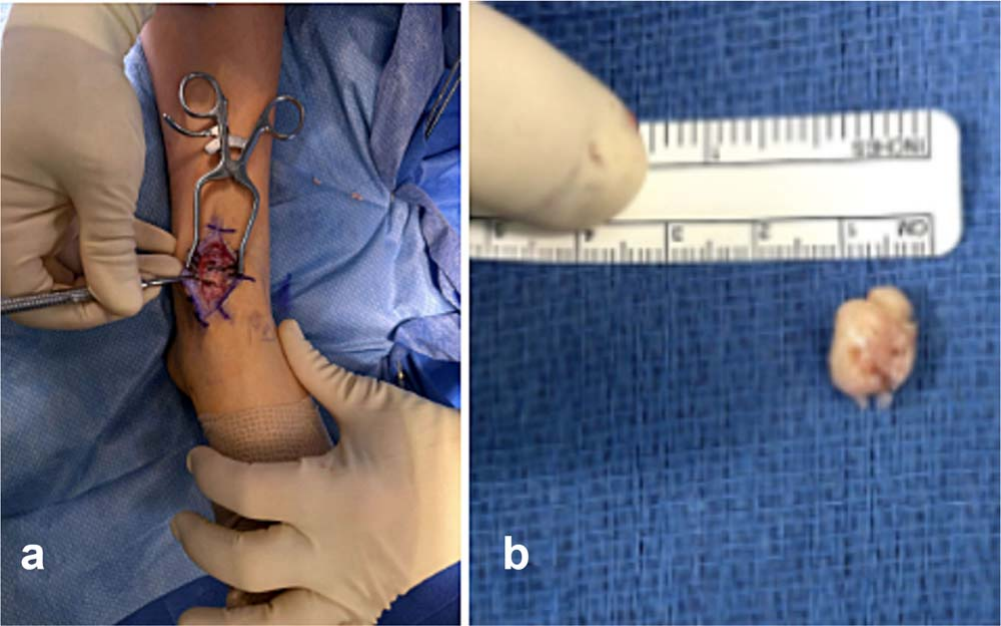
Intraoperative images from the third procedure demonstrating the talar osteochondroma in situ causing mechanical impingement of the ankle (a) and the excised 1-cm lesion with measurement (b).

**Figure 5:**
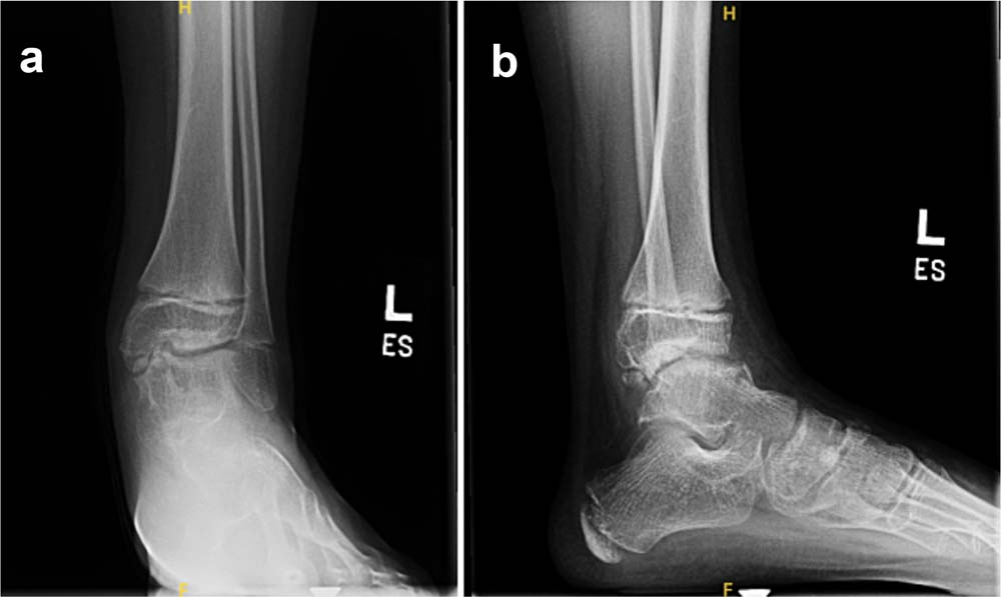
Postoperative AP (a) and lateral (b) radiographs of the left ankle demonstrating persistent irregularity of the distal tibia and talus with residual osteochondral changes following the third excision.

A third excision was performed through the same anteromedial approach. A single osteochondroma measuring ~1 cm was identified and excised from the subtalar joint (Fig. 4). Fluoroscopy confirmed complete removal and no additional loose bodies were identified. Postoperative radiographs demonstrated persistent irregularity of the distal tibia and talus with residual osteochondral changes consistent with postoperative remodeling (Fig. 5). No postoperative complications occurred. At 3-month follow-up in early 2026, the patient was recovering well and had begun structured physical therapy. Symptoms had improved compared with earlier in the disease course, although recurrence remains possible given continued skeletal immaturity.

## Discussion

DEH is an uncommon developmental disorder characterized by asymmetric epiphyseal osteochondral overgrowth with highly variable clinical behavior. Although surgical excision remains the primary treatment for symptomatic lesions or those causing joint deformity, outcomes are inconsistent and recurrence may occur. A review by Artioli *et al*. reported recurrence in ~9% of 70 patients following excision [2]. In contrast, our patient experienced two recurrences over a five-year period despite apparent complete excision confirmed intraoperatively, demonstrating a more aggressive pattern than typically reported.

The etiology of recurrence in DEH remains unclear. One proposed explanation is continued pathological cartilage proliferation in skeletally immature patients with open physes [12]. In this case, recurrence occurred at the talus on two separate occasions while the patient remained skeletally immature, supporting the possibility that abnormal epiphyseal growth may persist alongside normal skeletal development. Surgical intervention was therefore guided by clinical symptoms rather than radiographic progression, with surgery performed once functional limitations developed. However, even with careful resection and intraoperative fluoroscopy, complete removal of cartilaginous components may be difficult to achieve, and incomplete excision has been proposed as a cause of recurrence [13].

Repeated surgical intervention in skeletally immature patients must also be balanced against potential long-term complications. Persistent joint incongruity or stiffness following multiple procedures may predispose patients to early degenerative arthritis [14]. In severe cases, procedures such as tibiotalar or subtalar arthrodesis have been described as salvage options [15]. However, these procedures remain undesirable in young patients and are typically reserved for advanced deformity or refractory symptoms.

## Conclusion

DEH remains a challenging condition in pediatric orthopedics due to its unpredictable course and clinical rarity. This case demonstrates that recurrence may occur multiple times despite apparently complete surgical excision, particularly in skeletally immature patients with open physes. Management should prioritize preservation of joint anatomy while balancing the need for surgical intervention against the risks of repeated procedures. Shared decision making and counseling regarding the potential for recurrence are essential, as DEH should be managed as a chronic condition requiring longitudinal follow-up rather than a single surgically correctable pathology.

## References

[1] Smith EL, Raney EM, Matzkin EG et al. Trevor's disease: the clinical manifestations and treatment of dysplasia epiphysealis hemimelica. J Pediatr Orthop B 2007;16:297–302. 10.1097/BPB.0b013e328092563fS 17527110

[2] Artioli E, Mazzotti A, De Pellegrin M et al. Unveiling dysplasia epiphysealis hemimelica (Trevor's disease) in the foot and ankle: a systematic review. J Orthop 2024;52:49–54. 10.1016/j.jor.2024.02.036 38435317 PMC10901691

[3] Struijs PAA, Kerkhoffs GMMJ, Besselaar PP. Dysplasia epiphysealis hemimelica: a report of 3 cases. J Foot Ankle Surg 2012;51:620–6. 10.1053/j.jfas.2012.05.006 22819617

[4] Kantiwal P, Chawla S, Suhail A et al. Exceptionally extensive Trevor’s disease involving all four radial carpals presenting with gross wrist deformity. Cureus 2026;**18**:e101327. 10.7759/cureus.101327 PMC12892145 41684982

[5] Tyler PA, Rajeswaran G, Saifuddin A. Imaging of dysplasia epiphysealis hemimelica (Trevor's disease). Clin Radiol 2013;68:415–21. 10.1016/j.crad.2012.08.010T 23040212

[6] Ionescu A, Popescu B, Neagu O et al. Dysplasia epiphysealis hemimelica (Trevor’s disease) in children, two new cases: diagnosis, treatment, and literature review. Children 2021;8:907. 10.3390/children8100907 34682172 PMC8600412

[7] Goff T, Kan JH, Steiner RD et al. MR imaging of dysplasia epiphysealis hemimelica: bony and soft-tissue abnormalities. AJR Am J Roentgenol 1999;172:10063889. 10.2214/ajr.172.3.10063889 10063889

[8] Fairbank TJ . Dysplasia epiphysialis hemimelica. J Bone Joint Surg Br 1956;38-B:237–57. 10.1302/0301-620X.38B1.237 13295331

[9] Azouz EM, Slomic AM, Marton D et al. The variable manifestations of dysplasia epiphysealis hemimelica. Pediatr Radiol 1985;15:44–9. 10.1007/BF02387852 3969295

[10] Vogel T, Skuban T, Kirchhoff C et al. Dysplasia epiphysealis hemimelica of the distal ulna: a case report and review of the literature. Eur J Med Res 2009;14:272. 10.1186/2047-783X-14-6-272 19541588 PMC3352020

[11] Glick R, Khaldi L, Ptaszynski K et al. Dysplasia epiphysealis hemimelica (Trevor disease): a rare developmental disorder of bone mimicking osteochondroma of long bones. Hum Pathol 2007;38:1265–72. 10.1016/j.humpath.2007.01.017 17490719

[12] Kuo RS, Bellemore MC, Monsell FP et al. Dysplasia epiphysealis hemimelica: clinical features and management. J Pediatr Orthop 1998;18:543–8. 9661870

[13] Bahk WJ, Lee HY, Kang YK et al. Dysplasia epiphysealis hemimelica: radiographic and magnetic resonance imaging features and clinical outcome of complete and incomplete resection. Skeletal Radiol 2010;39:85–90. 19813010 10.1007/s00256-009-0803-x

[14] Ewalefo SO, Dombrowski M, Hirase T et al. Management of posttraumatic ankle arthritis: literature review. Curr Rev Musculoskelet Med 2018;11:546–57. 10.1007/s12178-018-9525-9E 30327933 PMC6220012

[15] Graviet S, Thomas J. Dysplasia epiphysealis hemimelica affecting the subtalar joint. J Am Podiatr Med Assoc 1994;84:580–2. 10.7547/87507315-84-11-580 7807388

